# Anti-tumorigenic and Platinum-Sensitizing Effects of Apolipoprotein A1 and Apolipoprotein A1 Mimetic Peptides in Ovarian Cancer

**DOI:** 10.3389/fphar.2018.01524

**Published:** 2019-01-28

**Authors:** Aline T. Marinho, Haonan Lu, Sofia A. Pereira, Emília Monteiro, Hani Gabra, Chiara Recchi

**Affiliations:** ^1^CEDOC Chronic Diseases Research Centre, NOVA Medical School, Faculdade de Ciências Médicas, Universidade NOVA de Lisboa, Lisbon, Portugal; ^2^Ovarian Cancer Action Research Centre, Imperial College London, London, United Kingdom

**Keywords:** apolipoprotein A1, ApoA1 mimetic peptides, ovarian cancer, platinum sensitization, invasiveness

## Abstract

**Objective:** Apolipoprotein A1 (ApoA1) is remarkably decreased in serum and ovarian tissues of ovarian cancer patients. ApoA1 and ApoA1 mimetic peptides can sequestrate pro-inflammatory phospholipids, some of which are known to activate a variety of oncogenic pathways. Besides, more intrinsic anti-tumorigenic properties, independent from interaction with lipids, have also been described for ApoA1. We aimed to disclose the effects of ApoA1 and a mimetic peptide on the malignant phenotype of ovarian cancer cells, particularly regarding cell viability, invasiveness and platinum sensitization.

**Methods:** Cells viability was assessed by MTS assay. Extracellular matrix invasion was assessed by transwell and spheroid invasion assays. Western blotting was performed to evaluate the effect of test compounds on intracellular pathways. Sensitization assays were performed *in vitro* and in the biologically relevant *in ovo* chorioallantoic membrane model.

**Results:** Both ApoA1 and the mimetic peptide, at a concentration of 100 μg/mL, were able to decrease the viability of SKOV3, CAOV3, and OVCAR3 cells (*p* < 0.05). The peptide at this concentration was not able to affect the viability of immortalized non-neoplastic ovarian cells (*p* > 0.05). ApoA1 decreased SKOV3 cells invasiveness at 300 μg/mL after 72 and 96 h of exposure (*p* < 0.05), while the ApoA1 mimetic peptide prevented cell invasion at 50 and 100 μg/mL (*p* < 0.01). Treatment with 100 μg/mL of ApoA1 mimetic peptide decreased Akt phosphorylation in SKOV3 cells (*p* < 0.01). Accordingly, treatment with increasing concentrations of the peptide sensitized SKOV3, OVCAR3 and CAOV3 cells to cisplatin. This synergistic effect was observed both *in vitro* and *in ovo*.

**Conclusions:** These results support the role of ApoA1 and ApoA1 mimetics as suppressors of ovarian tumorigenesis and as chemo-sensitising agents.

## Introduction

Ovarian cancer is the most deadly gynecological neoplastic disease among women worldwide (Coburn et al., 2017). Up to 85% of patients with epithelial ovarian cancer who achieve full remission after 1st line platinum-based chemotherapy will develop recurrent disease (Foley et al., 2013). The effectiveness of currently available treatments for this malignancy remains largely unsatisfactory and the 5-year survival rate is as low as 46% (Miller et al., 2016). For these reasons, new therapeutic strategies for ovarian cancer are urgently needed.

High density lipoprotein (HDL) particles are protein-rich lipoprotein complexes characterized by heterogeneous composition and function, with apolipoprotein A1 (ApoA1) being the major structural and functional component of the HDL proteome (Kontush et al., 2015). The best-known role of this apolipoprotein is in the maintenance of cholesterol homeostasis by promoting reverse cholesterol transport (Heinecke, 2012). However, HDL and ApoA1 have pleiotropic effects that substantiate the importance of these components in several other physiological functions (Bogan and Hennebold, 2010; Sriraman et al., 2010; Zamanian-Daryoush et al., 2013). For example, ApoA1 exerts an essential role in ovarian physiology and ovarian steroidogenesis (Bogan and Hennebold, 2010) and indeed the levels of this apolipoprotein are remarkably decreased in both serum and ovarian tissue of patients suffering from ovarian cancer (Stavnes et al., 2014; Wegdam et al., 2014). For this reason, ApoA1 was included in a panel of five biomarkers for assessing the likelihood of malignancy of an ovarian mass (Nolen and Lokshin, 2013). In this context, higher serum ApoA1 levels are associated with better prognosis and longer overall survival among patients suffering from ovarian cancer (Stavnes et al., 2014). The anti-tumorigenic properties of ApoA1 have been recently studied (Su et al., 2010; Ganapathy et al., 2012; Gao et al., 2012; Zamanian-Daryoush et al., 2013; Gkouskou et al., 2016) and it was found that ApoA1 and ApoA1 mimetic peptides can bind to pro-inflammatory pro-oncogenic phospholipids, namely to lysophosphatidic acid (LPA) (Van Lenten et al., 2008; Su et al., 2010; Yeh et al., 2016), whose plasma levels are increased in approximately 90% of all ovarian cancer patients (Bast et al., 2009). Importantly, LPA can activate a variety of cell signaling pathways with oncogenic potential, leading to increased survival, proliferation, motility, invasion, neoangiogenesis and chemoresistance (Li et al., 2009; Fan et al., 2015; Riaz et al., 2016). Furthermore, ApoA1 seems to have also other anti-tumorigenic properties, which are independent from LPA interaction and sequestration (Zamanian-Daryoush et al., 2013). The research work reported in the current paper was aimed at disclosing the effects of ApoA1 and an ApoA1 mimetic peptide (Ac-F^3,14^18A-NH_2_) on the malignant phenotype of ovarian cancer cells, particularly focusing on cell viability, invasiveness and the potential to synergize with platinum.

## Materials and Methods

### Cell Culture

SKOV3 cells (ATCC^®^HTB-77^TM^) were maintained in RPMI-1640 medium (Sigma-Aldrich, MO, United States) supplemented with 10% fetal bovine serum (FBS), 2 mM L-glutamine and 50 U/mL penicillin/streptomycin (Invitrogen, CA, United States). OVCAR3 cells (ATCC^®^HTB-161^TM^) were maintained in RPMI-1640 medium supplemented with 10% FBS, 2 mM L-glutamine, 50 U/mL penicillin/streptomycin and 0.01 mg/mL bovine insulin (Sigma-Aldrich, MO, United States). CAOV3 cells (ATCC^®^HTB-75^TM^) were maintained in DMEM medium (Sigma-Aldrich, MO, United States) supplemented with 10% FBS, 2 mM L-glutamine and 50 U/mL penicillin/streptomycin. SKOV3, OVCAR3, and CAOV3 cells were cultured at 37°C in a humidified atmosphere of 5% CO_2_. Immortalized non-neoplastic human ovarian surface epithelial cells (OSEC2) was kindly provided by Dr. Richard Edmondson (Newcastle University, United Kingdom). OSEC2 cells were kept in RPMI-1640 medium supplemented with 10% FBS, 2 mM L-glutamine and 50 U/mL penicillin/streptomycin, at 33°C in a humidified atmosphere of 5% CO_2_, as described (McKie et al., 2012). All cell lines were regularly tested to exclude mycoplasma contamination.

### Cell Viability and Caspase-Dependent Apoptosis Assay

SKOV3 cells were seeded in 96-well plates (5,000 cells/well) in full medium conditions and were allowed to attach for 24 h at 37°C. Cell medium was then changed to serum-free RPMI-1640. After overnight serum starvation, cells were incubated with increasing concentrations of ApoA1 (Sigma-Aldrich, MO, United States; 1–100 μg/mL) for 48 h. Mitochondrial viability was measured by CellTiter96^®^ AQueous One Solution Cell Proliferation assay (Promega, WI, United States). The results of cell viability for each condition were normalized to the control, which consisted of untreated cells exposed to vehicle only. Two independent experiments were performed, and each condition was run in triplicate. For the apoptosis assay, SKOV3 cells were seeded in 96-well white flat bottom plates (5,000 cells/well) in full medium conditions and were allowed to attach for 24 h at 37°C. Cell medium was then changed to serum-free RPMI-1640. After overnight serum starvation, cells were incubated with 100 μg/mL ApoA1 for 12 and 48 h. Caspase 3/7 activation was measured using the ApoTox-Glo^TM^ triplex assay (Promega, WI, United States). Caspase 3/7 activity was normalized to cell viability obtained for each condition. The results of caspase 3/7 activation were compared to the untreated control cells. Two independent experiments were performed, and each condition was run in triplicate.

The effect of the ApoA1 mimetic peptide (4F or Ac-F^3,14^18A-NH_2_; sequence: acetyl-DWFKAFYDKVAEKFKEAF-NH_2_; JPT Peptide Technologies, Berlin, Germany) on mitochondrial viability was investigated in SKOV3, OVCAR3, and CAOV3 cell lines. The toxicity of this peptide for non-neoplastic OSEC2 cells was also assessed. SKOV3, OVCAR3, and CAOV3 cells were seeded in 96-well plates (5,000 cells/well) in full medium conditions. Cells were allowed to attach for 24 h at 37°C. Cells were then kept in serum-free medium and, after overnight serum starvation, they were incubated with increasing concentrations of ApoA1 mimetic peptide (0.5–100 μg/mL)^1^ for 48 h. Similarly, OSEC2 cells (5,000 cells/well) were seeded in 96-well plates in full medium conditions. Cells were allowed to attach for 24 h at 33°C. Cell medium was changed to serum-free RPMI-1640 and after overnight serum starvation, cells were incubated with 100 μg/mL ApoA1 mimetic peptide for 48 h. The mitochondrial viability was measured by CellTiter96^®^ AQueous One Solution Cell Proliferation assay. The results of cell viability for each condition were normalized to the viability of untreated cells. Two independent experiments were performed for each cell line; each condition was run in triplicate.

### 3D Tumor Spheroid Invasion Assay

SKOV3 cells were cultured in ultra-low attachment round bottom 96-well plates, at a density of 5,000 cells/well in full medium conditions. Cells were kept at 37°C. After 3 days, the spheroids were serum-starved for 12 h. After this period of serum starvation, the spheroids were gently embedded in a mixture with equal volumes of serum-free RPMI-1640 and cold liquid growth factor reduced, phenol red-free, basement membrane Matrigel^®^ matrix (BD Biosciences, NJ, United States). Low concentration serum (0.3%) was added to stimulate invasion. ApoA1 was added to the mixture at a concentration of 300 μg/mL, while untreated spheroids exposed to vehicle only were used as controls. The invasion of extracellular matrix was assessed daily for 4 days, using an inverted microscope. Quantitative image analysis was performed with *ImageJ* software (National Institutes of Health, Bethesda, MD, United States). The invasion area was normalized to the spheroid area at time = 0. Two independent experiments were performed, each condition was run in triplicate.

### Transwell Migration Assay

SKOV3 cells were cultured in 6-well plates, at a cell density of 500,000 cells/well in RPMI-1640 supplemented with 10% FBS (full media conditions). Cells were allowed to attach for 24 h at 37°C in a humidified atmosphere of 5% CO_2_. Cells were then pre-treated with either 50 or 100 μg/mL ApoA1 mimetic peptide in serum free conditions. Untreated cells, exposed to vehicle only, were used as controls. After 24 h, 50,000 pre-treated and control cells were seeded in the top compartment of Matrigel^®^ coated 8 μm pores transwell culture inserts (Corning Life Sciences, Corning, NY, United States) in the presence of either 50 or 100 μg/mL ApoA1 mimetic peptide in serum free conditions. Control cells were again exposed to vehicle only. Complete medium containing 10% FBS was added to the bottom compartment of the plate as chemoattractant. The cells were then incubated for 2 days at 37°C in a humidified atmosphere of 5% CO_2_. Cells that had not migrated were removed from inside the transwell with a cotton swab. Cells were then fixed in cold methanol at -20°C for 5 min and stained with crystal violet for 10 min. The number of cells present in the bottom compartment of each well was then determined using an inverted microscope. Two independent experiments were performed, each condition was run in duplicate.

### Western Blotting

For western blot analysis SKOV3 cells were seeded in 12-well plates (60,000 cells/well) and allowed to attach during 24 h at 37°C. Cells were serum-starved overnight and then treated with the ApoA1 mimetic peptide at a concentration of 50 or 100 μg/mL, for 12 and 24 h in serum-free conditions, before cell lysis for 20 min on ice. Briefly, cells were washed with PBS prior to collection in RIPA buffer (Sigma-Aldrich, MO, United States) supplemented with protease inhibitor cocktail (Roche, Basel, Switzerland) and phosphatase inhibitor cocktail II (Calbiochem, San Diego, CA, United States) at the manufacturers’ recommended concentrations. Total protein concentration in the whole lysate was determined using the BCA Protein Assay Kit (Thermo Fisher Scientific, Waltham, MA, United States). Lysates were incubated at 100°C for 10 min and then separated into 10% sodium dodecyl sulphate polyacrylamide gel electrophoresis (SDS-PAGE). Gels were then transferred onto polyvinylidene difluoride membranes (Millipore, MA, United States). Membranes were blocked with 5% bovine serum albumin (BSA; Thermo Fisher Scientific, Waltham, MA, United States) in Tris-buffered saline polysorbate-20 (TBST) buffer. Primary antibodies were diluted in 5% BSA in TBST buffer as follows: 1:6,000 dilution for the calnexin antibody (ENZO Life Sciences, Farmingdale, NY, United States); 1:2,000 dilution for both phospho-p44/42 MAPK (ERK1/2) (Thr^202^/Tyr^204^) and p44/42 MAPK (ERK1/2) rabbit antibodies; 1:1,000 dilution for both the phospho-Akt Ser^473^ and the pan Akt rabbit antibodies (Cell Signaling Technology, MA, United States). Incubation with primary antibodies was performed at 4°C overnight. Horseradish peroxidase (HRP)-conjugated polyclonal goat anti-rabbit IgG (Agilent Technology, CA, United States) was employed at a 1:5,000 dilution in 1% BSA in TBST buffer, for incubation for 2 h at room temperature. Proteins were detected using Immobilon^TM^ Western Chemiluminescent HRP Substrate system (Millipore, MA, United States) and developed on X-ray film using a Kodak SRX2000 (Rochester, NY, United States) developer machine. Quantitative densitometry was performed using *ImageJ* software (National Institutes of Health, Bethesda, MD, United States).

### *In vitro* Cisplatin Sensitization Assay

SKOV3, OVCAR3, and CAOV3 cells were seeded in 96-well plates (5,000 cells/well) in full medium conditions. Cells were allowed to attach for 24 h at 37°C. Cells were then exposed to the ApoA1 mimetic peptide and cisplatin in full medium conditions for 72 h. Concentrations of the ApoA1 mimetic peptide ranged from 50 to 150 μg/mL, while cisplatin concentrations varied according to the sensitivity of each cell line. Mitochondrial viability was determined by the CellTiter96^®^ AQueous One Solution Cell Proliferation assay. The results for each condition were normalized to the control, which consisted of untreated cells. The viability of cells incubated with the peptide in combination with cisplatin was compared to the viability of cells exposed to cisplatin only. Two independent experiments were performed, including four replicates per condition.

The combination index (CI) was calculated for each concentration as the ratio between the isolated effect of both ApoA1 mimetic peptide and cisplatin and the combined effect of these compounds:

$$\mathrm{CI}\;=\;\frac{\left(\mathrm{100}\;-\;\%\;\mathrm{viable}\;{\mathrm{cells}}_{\mathrm{peptide}\;\mathrm{alone}}\right)\;+\;\left(\mathrm{100}\;-\;\%\;\mathrm{viable}\;{\mathrm{cells}}_{\mathrm{cisplatin}\;\mathrm{alone}}\right)}{\left(\mathrm{100}\;-\;\%\;\mathrm{viable}\;{\mathrm{cells}}_{\mathrm{peptide}\;\mathrm{and}\;\mathrm{cisplatin}\;}\right)}$$

A value of CI < 1 indicates that the combined effect of peptide and cisplatin is greater than the expected additive effect (synergy), while a value of CI > 1 results from a combined effect that is lesser than the expected additive effect (antagonism). Additionally, isoboles were plotted for each cell line, according to the Loewe additivity model for synergy/antagonism calculation (Foucquier and Guedj, 2015).

### *In ovo* Chicken Chorioallantoic Membrane Assay

All the procedures involving chicken embryos were performed in agreement with Community and national legislation (Directive 2010/63/EU; Animals Scientific Procedures Act 1986) and also in agreement with the Basel Declaration. The procedure received prior approval by the competent National Authority (project license: PPL70/7997; personal license: I1D60DB1A) and by Imperial College’s Animal Welfare and Ethical Review Body.

Fertilized chicken eggs (Henry Stewart & Co. Ltd., Norfolk, United Kingdom) were incubated at 37.5°C with 50% relative humidity for 3 days. On embryonic day 3, under sterile conditions, a small hole was pierced through the egg’s taglion using a 19-gauge needle and 2–3 mL of albumen were removed to decrease the volume inside the egg and to ensure that the chicken chorioallantoic membrane (CAM) is not damaged when the egg shell is opened. A 1 cm diameter window was opened on the egg’s shell to expose the CAM. The window was covered with sterile film and the eggs were incubated for 6 days. At embryonic day 9, xenografts were prepared by suspending 1 × 10^6^ green fluorescent protein (GFP)-transduced SKOV3 cells in 100 μL of Matrigel^®^. ApoA1 mimetic peptide and cisplatin were added to the cell suspension, at a concentration of 100 μg/mL and 15 μM, respectively. The compounds were added separately or in combination. Additionally, untreated cells were used as control. A medium to large blood vessel was then gently bruised using autoclaved round-bottom glass rods and the xenografts were topically inoculated onto this area. After inoculation, the egg shell window was covered with sterile tape and the eggs were placed back in the incubator for 7 days. Tumor dimensions were quantified at embryonic day 16 using Zeiss SteREO Discovery V8 microscope and the Zen 2.0 blue edition software (Oberkochen, Germany).

The CI was also calculated for the *in ovo* CAM assay, as the ratio between the isolated effects of 100 μg/mL ApoA1 mimetic peptide and 15 μM cisplatin, and the combined effect of these compounds as explained above:

$$\mathrm{CI}\;=\;\frac{\left(1\;-\;\mathrm{xenografts}\;{\mathrm{size}}_{\mathrm{peptide}\;\mathrm{alone}}\right)\;+\;\left(1\;-\;\mathrm{xenografts}\;{\mathrm{size}}_{\mathrm{cisplatin}\;\mathrm{alone}}\right)}{\left(1\;-\;\mathrm{xenografts}\;{\mathrm{size}}_{\mathrm{peptide}\;\mathrm{and}\;\mathrm{cisplatin}}\right)}$$

### Statistical Analysis

Statistical analysis was performed using GraphPad^®^Prism version 5.0 (GraphPad Software Inc., CA, United States). Data are presented as a percentage or as fold change compared to control. Data were analyzed by two-way ANOVA, Kruskal–Wallis, Mann–Whitney or Student’s *t*-test, as appropriate; a *p*-value < 0.05 was considered significant.

## Results

### Apolipoprotein A1 Decreases the Viability of Ovarian Cancer Cells

**Figure 1:**
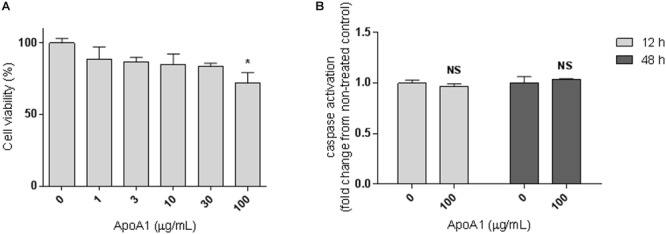
ApoA1 decreases the viability of ovarian cancer cells without affecting caspase activation. **(A)** SKOV3 cells were incubated with human ApoA1 up to a concentration of 100 μg/mL in serum-free conditions for 48 h. Untreated cells were exposed to vehicle only. ApoA1 treatment significantly decreased the viability of SKOV3 cells (Kruskal–Wallis test with Dunn’s multiple comparison post-test; *p* < 0.05). **(B)** SKOV3 cells were incubated with 100 μg/mL ApoA1 for 12 h (light gray bars) and 48 h (dark gray bars). Caspase 3/7 activation was quantified and normalized to the number of viable cells. No differences were observed in caspase activation between untreated cells and cells exposed to ApoA1. Differences were considered significant if *p* < 0.05. ^∗^*p* < 0.05.

In order to investigate whether human plasmatic ApoA1 affects ovarian cancer cells viability, we analyzed the effect of ApoA1, added to the extracellular medium, on the viability of SKOV3 cells. These assays were performed in serum-free conditions to isolate the effect of human ApoA1, avoiding interferences with bovine ApoA1 and the variety of growth factors present in the serum for cell medium supplementation. Under these conditions, 48-h treatment with 100 μg/mL ApoA1 reduced the viability of SKOV3 cells by 28% (Figure 1A; Kruskal–Wallis with Dunn’s post-test; *p* < 0.05). This effect of ApoA1 on SKOV3 cells viability was independent of caspase 3/7 activation, as observed after 12 and 48 h of incubation with ApoA1 (Figure 1B; two-way ANOVA; *p* > 0.05).

### Apolipoprotein A1 Reduces the Ability of Ovarian Cancer Cells to Invade the Extracellular Matrix

**Figure 2:**
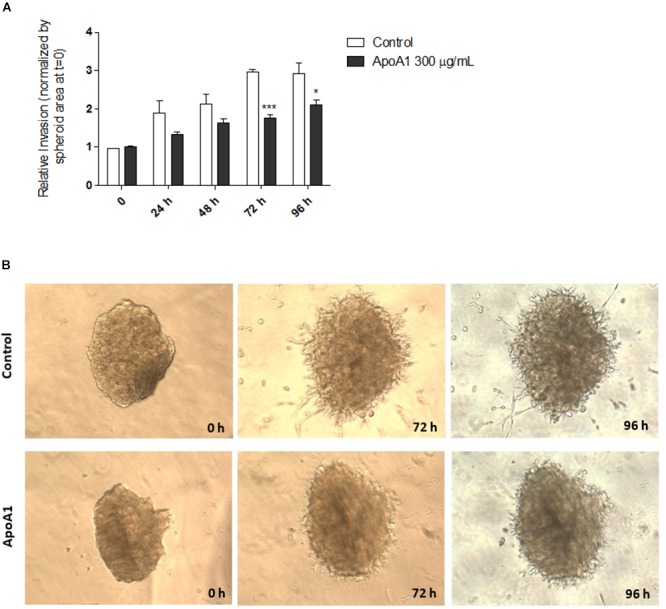
ApoA1 decreases the ability of ovarian cancer cells to invade the extracellular matrix. The 3D tumor spheroid invasion assay was performed in SKOV3 spheroids exposed to ApoA1 at a concentration of 300 μg/mL, and in unexposed spheroids (control). Invasion of extracellular matrix was assessed for up to 96 h. Exposure to ApoA1 significantly decreased the ability of SKOV3 cells to invade the extracellular matrix after 72 h (Two-way ANOVA; *p* < 0.001) and 96 h (*p* < 0.05). In **(A)**, the relative invasion normalized to the spheroid area at time = 0 is plotted for ApoA1-treated and untreated spheroids. In **(B)**, it is shown two spheroids, representatives of the ApoA1-treated and control conditions, at the beginning of the experiment (time = 0) and during the course of experiment (72 and 96 h). Differences were considered significant if *p* < 0.05. ^∗^*p* < 0.05, ^∗∗∗^*p* < 0.001.

An important feature of cancer cells is the ability to invade the extracellular matrix in order to metastasise to different organs. Therefore, the effect of ApoA1 exposure on the ability of SKOV3 cells to invade the extracellular matrix was investigated in a 3D tumor spheroid invasion assay. This assay was performed in spheroids unexposed (control) and exposed to 300 μg/mL ApoA1 (Figure 2). Invasion of the extracellular matrix was assessed daily, for 4 days (96 h), and the invasion area for each day was normalized to the spheroid area at time = 0. As shown in Figure 2, ApoA1 treatment significantly decreased the ability of SKOV3 cells to invade the extracellular matrix after 72 h (Two-way ANOVA; control: 2.97 ± 0.06; ApoA1: 1.76 ± 0.09; *p* < 0.001) and 96 h of treatment (control: 2.93 ± 0.28; ApoA1: 2.11 ± 0.13; *p* < 0.05).

### The ApoA1 Mimetic Peptide Decreases the Viability of Ovarian Cancer Cells

**Figure 3:**
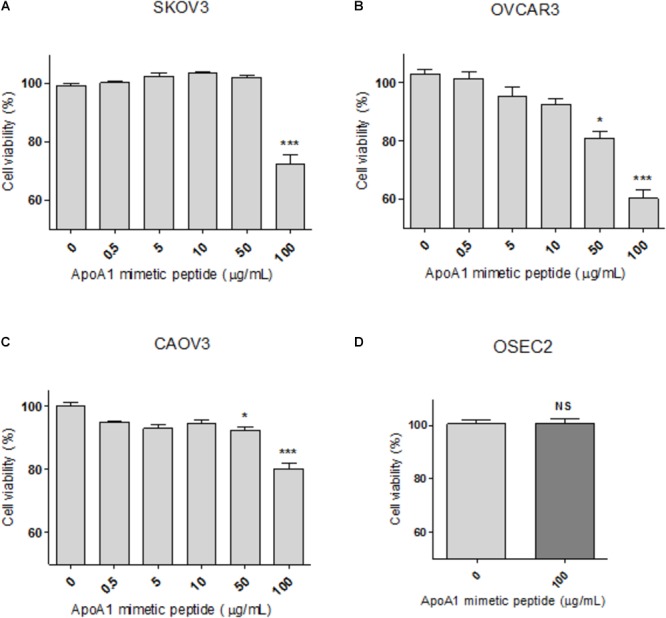
The ApoA1 mimetic peptide decreases the viability of ovarian cancer cells without affecting non-neoplastic ovarian cells. SKOV3 **(A)**, OVCAR3 **(B)**, and CAOV3 **(C)** cells were incubated with the ApoA1 mimetic peptide 4F up to a concentration of 100 μg/mL, in serum-free conditions for 48 h. Untreated cells were exposed to vehicle only. Exposure to the highest concentration of the peptide decreased the viability of all the three cell lines (Kruskal–Wallis test with Dunn’s multiple comparison post-test; *p* < 0.001). Immortalized non-neoplastic human ovarian surface epithelia cells (OSEC2; **D**) were exposed to the highest concentration of peptide (100 μg/mL) used in the viability assay for the ovarian cancer cell lines. At this concentration the peptide did not affect the viability of OSEC2 cells. Differences were considered significant if *p* < 0.05. ^∗^*p* < 0.05, ^∗∗∗^*p* < 0.001.

Synthetic ApoA1 mimetic peptides are able to effectively replicate several functional features of ApoA1, therefore in the current study the 4F ApoA1 mimetic was employed as a surrogate for ApoA1. This peptide was tested in three different ovarian cancer cell lines, SKOV3, OVCAR3, and CAOV3 cells. The viability of cells treated with 100 μg/mL 4F was reduced by 28, 40 and 20%, for SKOV3, OVCAR3, and CAOV3 cells, respectively (Figure 3A–C; Kruskal–Wallis with Dunn’s post-test; *p* < 0.001 for all cell lines). The peptide at a concentration of 50 μg/mL also reduced the viability of OVCAR3 and CAOV3 cells (Figure 3B,C; *p* < 0.05 for both cell lines). As shown in Figure 3, OVCAR3 cells appear to be the most sensitive to the ApoA1 mimetic peptide. Importantly, the ApoA1 mimetic peptide did not affect the viability of non-neoplastic ovarian surface epithelial OSEC2 cells (Figure 3D; Mann–Whitney, *p* > 0.05).

### The ApoA1 Mimetic Peptide Prevents Cancer Cell Invasion

**Figure 4:**
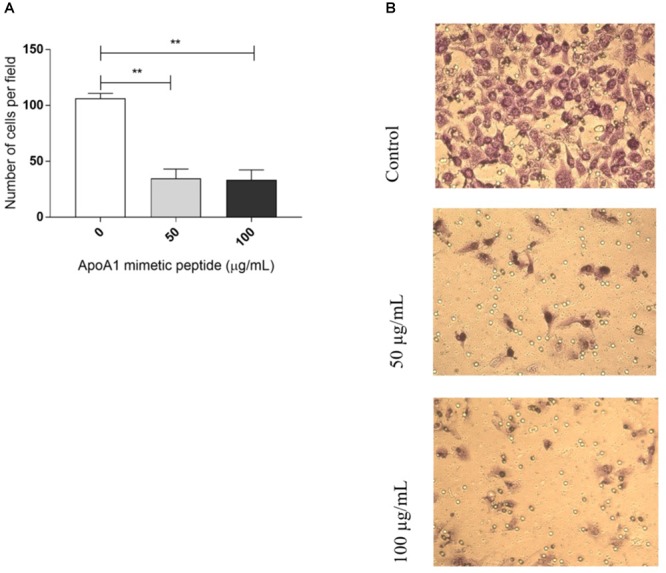
The ApoA1 mimetic peptide hampers cancer cells invasion. A transwell migration assay was performed with SKOV3 cells exposed to the ApoA1 mimetic peptide at a concentration of 50 and 100 μg/mL, and with unexposed SKOV3 cells (control). Exposure to the ApoA1 mimetic peptide at both concentrations significantly decreased the ability of SKOV3 cells to migrate and invade, comparatively to unexposed cells (Kruskal–Wallis test with Dunn’s post-test; control vs. 50 μg/mL ApoA1 mimetic peptide: *p* = 0.0020; control vs. 100 μg/mL ApoA1 mimetic peptide: *p* = 0.0016). In **(A)**, the number of cells per field is plotted for each condition. In **(B)**, it is shown three images representatives of each experimental condition. Differences were considered significant if *p* < 0.05. ^∗∗^*p* < 0.001.

Cancer cell migration and invasion are recognized as essential steps in the cascade of events that culminate in the establishment of a metastatic tumor. The effect of the ApoA1 mimetic peptide, at a concentration of 50 and 100 μg/mL, on the transmigration ability of SKOV3 cells was assessed in a transwell migration assay, employing Matrigel^®^ coated 8 μm pore transwells and FBS as chemoattractant. As shown in Figure 4A,B, the treatment with 50 or 100 μg/mL of ApoA1 mimetic peptide was able to prevent ovarian cancer cell invasion, as the number of cells that invaded the Matrigel and reached the bottom compartment of the plate was significantly lower comparatively to untreated cells (Kruskal–Wallis with Dunn’s post-test; control: 105.5 [93.8–119.8]; 50 μg/mL treatment: 29 [12–60.2]; control vs. 50 μg/mL treatment: *p* = 0.0020; 100 μg/mL treatment: 30 [13–61]; control vs. 100 μg/mL treatment: *p* = 0.0016).

### The ApoA1 Mimetic Peptide Strongly Affects Akt Phosphorylation

**Figure 5:**
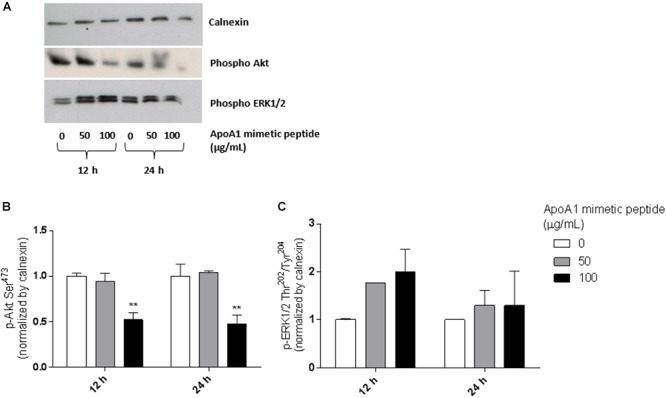
The ApoA1 mimetic peptide strongly suppresses Akt signaling in ovarian cancer cells. After overnight serum starvation, SKOV3 cells were treated with the ApoA1 mimetic peptide (50 or 100 μg/mL). Untreated SKOV3 cells were used as control. Cell lysates were collected 12 or 24 h after treatment and subjected to western blot analysis. Calnexin was the loading control for these experiments. **(A)** Western blot for phospho Akt (Ser^473^) and phospho ERK1/2 (Thr^202^/Tyr^204^); **(B)** densitometry for Akt Ser^473^ phosphorylation; **(C)** densitometry for ERK1/2 Thr^202^/Tyr^204^ phosphorylation. The ApoA1 mimetic peptide strongly decreased phosphorylation of Akt at Ser^473^ after 12 h (two-way ANOVA; *p* < 0.01) and 24 h (*p* < 0.01) of exposure with the highest concentration of peptide (100 μg/mL). Differences were considered significant if *p* < 0.05. ^∗∗^*p* < 0.001.

To better understand the mechanism behind the observed reduction in viability, we investigated whether the incubation with the ApoA1 mimetic peptide would affect pro-survival pathways such as the Akt and ERK1/2 signaling cascades in SKOV3 cells. As demonstrated in Figure 5, SKOV3 cells treated with 100 μg/mL ApoA1 mimetic peptide showed a decrease of approximately 50% in Akt Ser^473^ phosphorylation after 12 h (Figure 5A,B; two-way ANOVA; 0.52 ± 0.08; *p* < 0.01) or 24 h (0.48 ± 0.10; *p* < 0.01) of exposure. Despite not reaching statistical significance, a trend for increased phosphorylation of ERK1/2 at Thr^202^/Tyr^204^ upon treatment with the peptide was also observed. Nevertheless, this effect on ERK phosphorylation was more pronounced after 12 h of exposure, being reduced after 24 h of treatment (Figure 5A,C). No significant changes were observed regarding total Akt and total ERK1/2 levels (Supplementary Figure S1). These results support the hypothesis that the ApoA1 mimetic peptide could reduce cancer cell survival by preferentially affecting Akt signaling. Since the activation of the Akt pathway is key for the development of resistance to cisplatin in ovarian cancer patients (24), these results prompted us to evaluate the effect of this peptide on the sensitization of ovarian cancer cells to cisplatin.

### The ApoA1 Mimetic Peptide Sensitizes Ovarian Cancer Cells to Cisplatin

**Figure 6:**
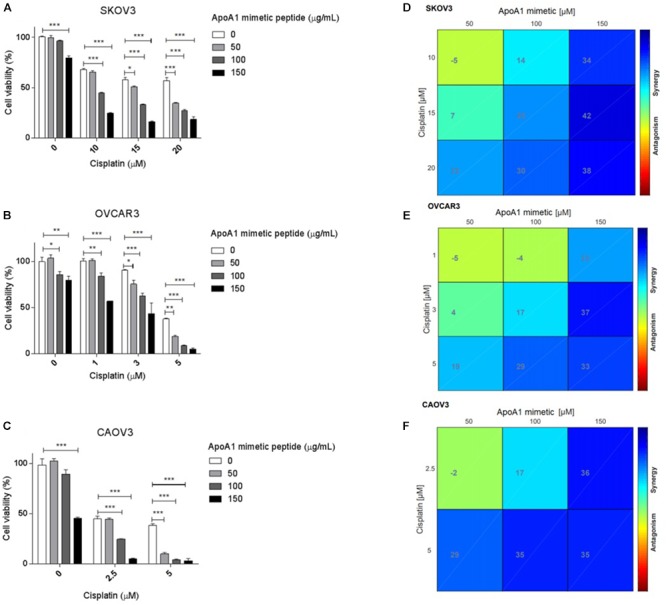
The ApoA1 mimetic peptide sensitizes ovarian cancer cells to cisplatin. SKOV3, OVCAR3, and CAOV3 cells were co-incubated with increasing concentrations of ApoA1 mimetic peptide, up to 150 μg/mL, and increasing concentrations of cisplatin, according to the sensitivity of each cell line. Exposure to the peptide and cisplatin was performed in full medium conditions. After 72 h, mitochondrial cell viability was assessed. Two-way ANOVA was performed as statistical test **(A–C)**. ^∗^*p* < 0.05; ^∗∗^*p* < 0.01; ^∗∗∗^*p* < 0.001. Isoboles for assessing synergistic interactions between the ApoA1 mimetic peptide and cisplatin were also plotted **(D–F)**, according to the Loewe additivity model for synergy/antagonism calculation. On the horizontal axis of the isoboles, the different concentrations of ApoA1 mimetic peptide employed in the *in vitro* sensitization assay are represented, while in the vertical axis it is represented the concentrations of cisplatin. The intersections represent the combined effect between the peptide and cisplatin. The color gradient ranges from dark blue (strong synergistic effect) to dark red (strong antagonistic effect). From the isoboles analysis, it is possible to identify synergies for different concentrations of ApoA1 mimetic peptide and cisplatin.

To investigate the ability of the ApoA1 mimetic peptide to induce the sensitization of ovarian cancer cells to platinum, we exposed SKOV3, OVCAR3, and CAOV3 cells to different concentrations of the peptide and cisplatin, and analyzed cell viability for each condition. The results presented in Figure 6 show that, in full medium conditions, the ApoA1 mimetic peptide sensitizes all the three ovarian cancer cell lines tested to cisplatin. In particular, the viability for SKOV3 cells exposed to 15 μM cisplatin only, with no peptide, was approximately 58%; while SKOV3 cells exposed to the same concentration of cisplatin but in combination with increasing concentrations of the peptide – 50, 100, and 150 μg/mL – presented a cell viability of 50% (Two-way ANOVA; *p* < 0.05), 33% (*p* < 0.001) and 16% (*p* < 0.001), respectively. Likewise, the viability of OVCAR3 cells was significantly reduced upon concomitant treatment with cisplatin and the ApoA1 mimetic peptide. For this cell line, treatment with 3 μM cisplatin resulted in approximately 90% cell viability; however, co-incubation with 50, 100, or 150 μg/mL of 4F peptide caused a significant decrease in cell viability, to 75% (*p* < 0.05), 62% (*p* < 0.001), and 43% (*p* < 0.001), respectively. Similarly, CAOV3 cells treated with 2.5 μM cisplatin reached a cell viability of 44%, which, in combination with 100 and 150 μg/mL of the ApoA1 mimetic peptide, significantly decreased to 24% (*p* < 0.001) and 5% (*p* < 0.001), respectively (Figure 6A–C).

The CI for each cell line and for each concentration of ApoA1 mimetic peptide and cisplatin is presented in Table 1. The isoboles for synergy/antagonism calculations are presented in Figure 6D–F. Resorting to both models, it was possible to identify synergistic interactions between the ApoA1 mimetic peptide and cisplatin, at different concentrations and for all the three cell lines tested.

**Table 1:** Combination index (CI) for cisplatin and the ApoA1 mimetic peptide *in vitro* and *in ovo*.

Cell line	Cisplatin concentration (μM)	Peptide concentration (μg/mL)	CI
SKOV3 (*in vitro*)	10	50	0.98
		100	0.65
		150	0.69
	15	50	0.88
		100	0.68
		150	0.74
	20	50	0.68
		100	0.64
		150	0.78
OVCAR3 (*in vitro*)	1	50	1.20
		100	0.87
		150	0.45
	3	50	0.41
		100	0.64
		150	0.52
	5	50	0.72
		100	0.84
		150	0.87
CAOV3 (*in vitro*)	2.5	50	0.97
		100	0.88
		150	1.10
	5	50	0.66
		100	0.75
		150	1.21
GFP-SKOV3 xenografts (*in ovo* CAM assay)	15	100	0.52

*The CI was calculated as the ratio between the isolated effects of ApoA1 mimetic peptide and cisplatin and the combined effect of these compounds, for SKOV3, OVCAR3, and CAOV3 cells exposed in vitro and also for GFP-transduced SKOV3 xenografts exposed to the ApoA1 mimetic peptide and cisplatin in the in ovo chicken CAM assay. A CI value below 1 represents a synergistic effect*.

### Effect of the ApoA1 Mimetic Peptide on Cisplatin Sensitization in the Chicken Chorioallantoic Membrane Model

To investigate the effect of the ApoA1 mimetic peptide in terms of cisplatin sensitization in a more biologically relevant system, we used the CAM assay. This *in ovo* model allows the engrafting of cancer cells on the surface of the CAM, a well-vascularised extra-embryonic tissue located underneath the eggshell, in embryonated eggs. We inoculated onto the chicken CAM GFP-transduced SKOV3 cell xenografts, exposed *in ovo* to cisplatin and ApoA1 mimetic peptide, separately or in combination.

**Figure 7:**
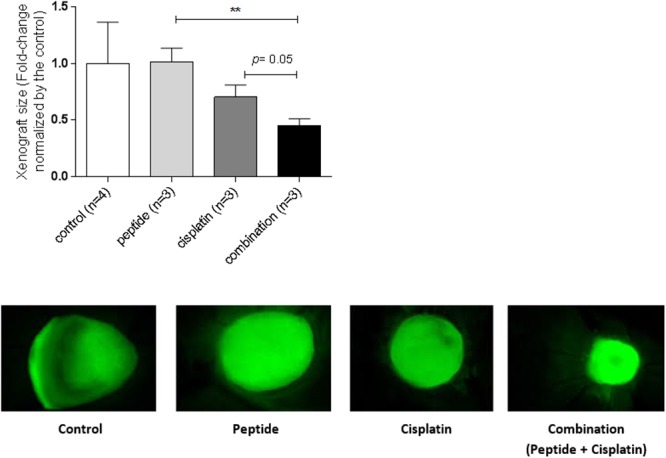
The ApoA1 mimetic peptide sensitizes ovarian cancer cells to cisplatin in the biologically relevant *in ovo* CAM model. SKOV3 xenografts were topically inoculated on the CAM, left untreated (control) or treated with the ApoA1 mimetic peptide (100 μg/mL), cisplatin (15 μM) or with the combination of both (100 μg/mL peptide plus 15 μM cisplatin). Xenograft size was calculated as the area of the fluorescent tumor relative to background. Student’s *t*-test was performed after testing the data normality by the Shapiro–Wilk normality test. Differences were considered significant if *p* < 0.05. ^∗∗^*p* < 0.01.

While cisplatin alone had a modest effect on the tumor size, we did not observe any effect of the peptide alone on the growth of the xenografts (Figure 7). However, the combination of the peptide and cisplatin had a more dramatic effect on tumor growth and the size of these xenografts was significantly smaller than those exposed to the peptide alone (Student’s *t*; peptide: 1.0 ± 0.12; combination: 0.45 ± 0.06; *p* = 0.007) or to cisplatin alone (cisplatin: 0.70 ± 0.11; combination: 0.45 ± 0.06; *p* = 0.05) (Figure 7). Besides, the CI for the *in ovo* CAM assay was 0.52 (Table 1), indicating that the combination of peptide and cisplatin is synergistic in this biologically relevant model. Therefore, the *in ovo* findings substantiate the *in vitro* results demonstrating the ability of the ApoA1 mimetic to induce cisplatin sensitization in ovarian cancer cells.

## Discussion

In the current study, we demonstrated the effect of ApoA1 and the ApoA1 mimetic peptide 4F as possible suppressors of ovarian tumorigenesis. In particular, we observed that these compounds can affect viability, inhibit invasion of the extracellular matrix and induce chemosensitisation in a panel of ovarian cancer cell lines.

The anti-tumorigenic properties of both ApoA1 and ApoA1 mimetics have been recently explored in different types of malignancies, including melanoma (Zamanian-Daryoush et al., 2013), colorectal (Gkouskou et al., 2016), and ovarian cancer (Su et al., 2010; Gao et al., 2011, 2012, Ganapathy et al., 2012). For instance, transgenic animal models overexpressing human ApoA1 showed decreased tumor development when inoculated with melanoma and lung cancer cells, while ApoA1 knockout had the opposite effect (Zamanian-Daryoush et al., 2013). Importantly, subcutaneous injection of human ApoA1 decreased tumor burden, prevented metastatic growth and improved survival of mice previously inoculated with melanoma cells, after the establishment of palpable tumors and metastasis (Zamanian-Daryoush et al., 2013), arguing for the potential therapeutic applications of ApoA1 for cancer at more advanced stages. Moreover, ApoA1 knockout in a rodent model of colitis-induced colorectal carcinogenesis was associated to exacerbated pathological and inflammatory features and also to higher proliferative index (Gkouskou et al., 2016). Relatively to ovarian malignancies, overexpression of human ApoA1 also decreased tumor volume, prevented metastatic growth and increased survival in a transgenic mouse model of ovarian cancer (Su et al., 2010). The anti-tumorigenic mechanisms attributed to ApoA1 are related to the modulation of the tumor microenvironment, the inhibition of tumor neoangiogenesis, the regulation of inflammatory signaling [e.g., through the signal transducer and activator of transcription 3 (STAT3) pathway] and to decreased levels of pro-invasion factors, such as matrix metalloproteinase-9 (MMP-9) (Zamanian-Daryoush et al., 2013; Gkouskou et al., 2016; Jia et al., 2017). Additionally, ApoA1 was demonstrated to decrease the viability of gastric adenocarcinoma cells *in vitro*, by abrogating the proliferation induced by LPA (Yeh et al., 2016), a pro-inflammatory pro-carcinogenic lysophospholipid that is crucially involved in the modulation of several intracellular pathways also contributing to ovarian cancer progression (Fang et al., 2002; Bast et al., 2009; Li et al., 2009; Fan et al., 2015; Riaz et al., 2016; Ottevanger, 2017). Therefore, the inhibitory effect of ApoA1 on these multiple factors could similarly contribute to the anti-tumorigenic phenotypes we have demonstrated here.

Besides ApoA1, some ApoA1 mimetic peptides have also been explored for their anti-tumorigenic properties. ApoA1 mimetics are synthetic amphipathic α-helical peptides that can mimic the structure of an ApoA1 α-helix and replicate, at least to some extent, several aspects of ApoA1 functionality (Datta et al., 2001). However, there are some functional differences between ApoA1 and its mimetic peptides. For instance, ApoA1 mimetic peptides show limited ability to activate the lecithin–cholesterol acyltransferase (LCAT), unlike ApoA1 which can fully activate this enzyme (Datta et al., 2001); in contrast, ApoA1 mimetic peptides can bind to pro-inflammatory and oxidized lipids, including LPA, more effectively than ApoA1 (Van Lenten et al., 2008; Su et al., 2010). Regarding the effect of ApoA1 mimetics in ovarian cancer, Su et al. (2010) reported the beneficial effect of two peptides, 4F and 5F (Ac-F^11,14,17^18A-NH_2_), in terms of tumor development *in vivo*, after subcutaneous and intraperitoneal injection of an epithelial ovarian cancer cell line in mice. The mechanisms implicated in this effect were related to the binding and sequestration of LPA and possibly other inflammatory lipids, consequently leading to a reduction in cellular oxidative stress and inhibition of pro-angiogenic signaling [e.g., hypoxia-inducible factor (HIF)-1-dependendent transcription of vascular endothelial growth factor (VEGF)] (Saunders et al., 2010; Su et al., 2010; Gao et al., 2011, 2012; Ganapathy et al., 2012). In regard to the anti-tumorigenic effects reported in the present work, the involvement of LPA sequestration seems plausible due to the high-affinity between 4F and LPA (Van Lenten et al., 2008; Su et al., 2010) and also considering that all three ovarian cancer cell lines employed here, unlike normal ovarian epithelial cells, produce LPA as an autocrine/paracrine factor (Chou et al., 2004). Furthermore, HIF-1-induced VEGF expression can lead to increased Akt phosphorylation at Ser^473^ in ovarian granulosa cells and in ovarian cancer cells (Trinh et al., 2009; Shiratsuki et al., 2016). Additionally, cholesterol depletion in membrane lipid rafts, promoted by both ApoA1 and the ApoA1 mimetic peptides, can inhibit Akt signaling (Iqbal et al., 2016). However, further studies need to be conducted to clarify the involvement and contribution of LPA sequestration or signaling modulation in lipid rafts to the effects mediated by ApoA1 or its mimetic peptides that we report here.

Importantly, a large body of evidence demonstrates hyperactivation of PI3K/Akt kinases in several malignancies (Stronach et al., 2011; Cheung and Testa, 2013; Mabuchi et al., 2015). This pathway not only contributes to ovarian cancer development and tumorigenesis but has also been implicated in the mechanism of chemoresistance of ovarian cancer cells to platinum and other drugs, such as taxanes and doxorubicin (Blagden and Gabra, 2009; Brasseur et al., 2017). Therefore, targeting Akt signaling has become an appealing strategy for overcoming chemoresistance in ovarian malignancies (Blagden and Gabra, 2009; Stronach et al., 2011; Cheraghchi-Bashi et al., 2015). Here we demonstrated that an ApoA1 mimetic peptide, employed at higher concentrations than the ones reported in previous studies (Su et al., 2010; Gao et al., 2011, 2012; Ganapathy et al., 2012), can affect Akt signaling and cancer cells sensitivity to platinum. Although more studies are needed to assess the safety and tolerability of ApoA1 and ApoA1 mimetic peptides at relevant doses for the anti-tumorigenic and chemosensitising effect, the potential translational application of this therapeutic approach is particularly promising (Foley et al., 2013; Miller et al., 2016) and the use of ApoA1 or a mimetic peptide in combination with conventional cytotoxic drugs might bring real benefits to women affected by ovarian cancer.

## Author Contributions

AM and HL carried out the experiments. AM, SP, and CR wrote the manuscript. AM, HL, SP, EM, HG, and CR contributed to the interpretation of the results and to the final version of the manuscript. All the authors provided essential feedback to the research work.

## Conflict of Interest Statement

HG (Head of the Clinical Discovery Unit in Early Clinical Development within the iMED Biotech Unit at AstraZeneca) reports financial relationships with AstraZeneca, outside the scope of the submitted work. The remaining authors declare that the research was conducted in the absence of any commercial or financial relationships that could be construed as a potential conflict of interest.

## Footnotes

1 100 μg/mL of ApoA1 corresponds to approximately 3.5 μM of protein (ApoA1 molecular weight: 28300 g/mol), while 100 μg/mL of the ApoA1 mimetic peptide corresponds to 43 μM of peptide (4F peptide molecular weight: 2310.62 g/mol).

## Supplementary Material

Figure S1 The apoA1 mimetic peptide does not affect the levels of pan Akt or total ERK1/2. SKOV3 cells were treated with the ApoA1 mimetic peptide (50 or 100 μg/mL) in serum free conditions. Unexposed SKOV3 cells were used as control. Cell lysates were collected 12 or 24 h after treatment and subjected to western blot analysis. Calnexin was employed as the loading control. **(A)** Densitometry for Pan Akt; **(B)** densitometry for total ERK1/2.
