# Supplementary figures and images for: Anti-tumorigenic and Platinum-Sensitizing Effects of Apolipoprotein A1 and Apolipoprotein A1 Mimetic Peptides in Ovarian Cancer
Source: Front Pharmacol. 2019 Jan 28;9:1524. doi: 10.3389/fphar.2018.01524 (PMC6360149; doi:10.3389/fphar.2018.01524)

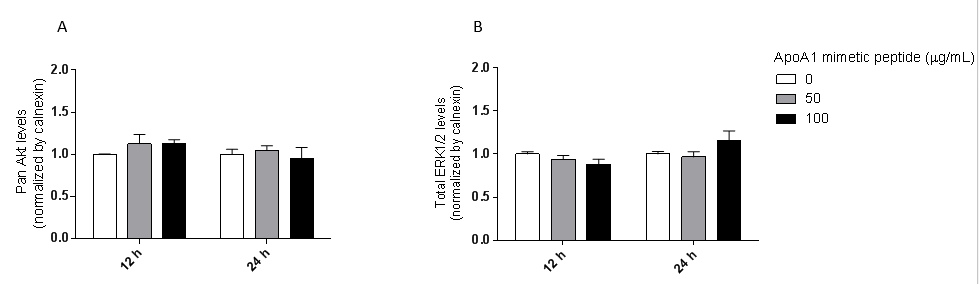

Supplement: Figure S1 — The apoA1 mimetic peptide does not affect the levels of pan Akt or total ERK1/2. SKOV3 cells were treated with the ApoA1 mimetic peptide (50 or 100 μg/mL) in serum free conditions. Unexposed SKOV3 cells were used as control. Cell lysates were collected 12 or 24 h after treatment and subjected to western blot analysis. Calnexin was employed as the loading control. (A) Densitometry for Pan Akt; (B) densitometry for total ERK1/2. [file Image_1.TIF]
